# The Metabolic Factor Kynurenic Acid of Kynurenine Pathway Predicts Major Depressive Disorder

**DOI:** 10.3389/fpsyt.2018.00552

**Published:** 2018-11-19

**Authors:** Hongye Liu, Lei Ding, Huifeng Zhang, David Mellor, Haiyan Wu, Dongmei Zhao, Chuangxin Wu, Zhiguang Lin, Jiaojian Yuan, Daihui Peng

**Affiliations:** ^1^Shanghai Mental Health Center, School of Medicine, Shanghai Jiao Tong University, Shanghai, China; ^2^School of Psychology, Deakin University, Melbourne, VIC, Australia; ^3^Instrumental Analysis Center, Shanghai Jiao Tong University, Shanghai, China

**Keywords:** major depressive disorder, kynurenine pathway, quinolinic acid, kynurenic acid, mass spectrometry

## Abstract

**Background:** Metabolic factors in the kynurenine pathway (KP) have been widely accepted as being a major mechanism in Major Depressive Disorder (MDD). However, the effects of these metabolites on the degree and pattern of MDD are still poorly understood, partly due to the elusiveness of the level of metabolites when diagnosing depression. This study aimed to explore a novel diagnostic method analyzing peripheral blood with mass spectrometry to assess metabolites from KP in patients with MDD and Bipolar Depression (BD).

**Methods:** Thirty-three patients with MDD, 20 patients with BD, and 23 healthy control participants were enrolled Metabolic factors of KP from plasma including tryptophan (TRP), kynurenine (KYN), kynurenic acid (KYNA), and quinolinic acid (QUIN) were analyzed by UPLC-3Q-MS, and levels compared across three groups. Correlation between HAMD scores and metabolite levels conducted. Receiver operating characteristic (ROC) curve was used to determine the diagnostic value of metabolic factors in MDD.

**Results:** Levels of KYNA, QUIN, KYNA/QUIN, and KYNA/KYN were statistically different across the three groups (*P* < 0.05); HAMD scores and TRP, KYN, KYNA/QUIN levels were negatively correlated in the MDD group (*r* = −0.633, −0.477, −0.418, *P* < 0.05); Accuracy of KYNA diagnosing MDD was 82.5% with the optimal diagnostic value being 15.48 ng/ml. Diagnostic accuracy was increased to 83.6% when KYNA and QUIN levels were used in combination.

**Conclusion:** This results indicate that metabolic factors of KP play a crucial role in the occurrence and development of MDD, supporting the metabolic imbalance hypothesis of MDD. Furthermore, our study also provides a new diagnostic method to study MDD based on plasma KYNA level, and suggests that KYNA would be a potential biomarker in diagnosing depression patients.

## Introduction

Major depressive disorder (MDD) is a serious mental disorder with high rates of morbidity, recurrence, and disability (1–3) that is associated with genetic factors, psychological factors and atypical brain structure or function (4, 5). Despite differences in pathogenesis, genetics, pathophysiology, etc., bipolar depression (BD) can easily be misdiagnosed as a subtype of MDD (6) due to its parallel clinical characteristic (7). It is important that these disorders are differentially diagnosed to ensure that relevant treatments are initiated, and to assist in this innovative approaches are critical.

**Figure 1:**
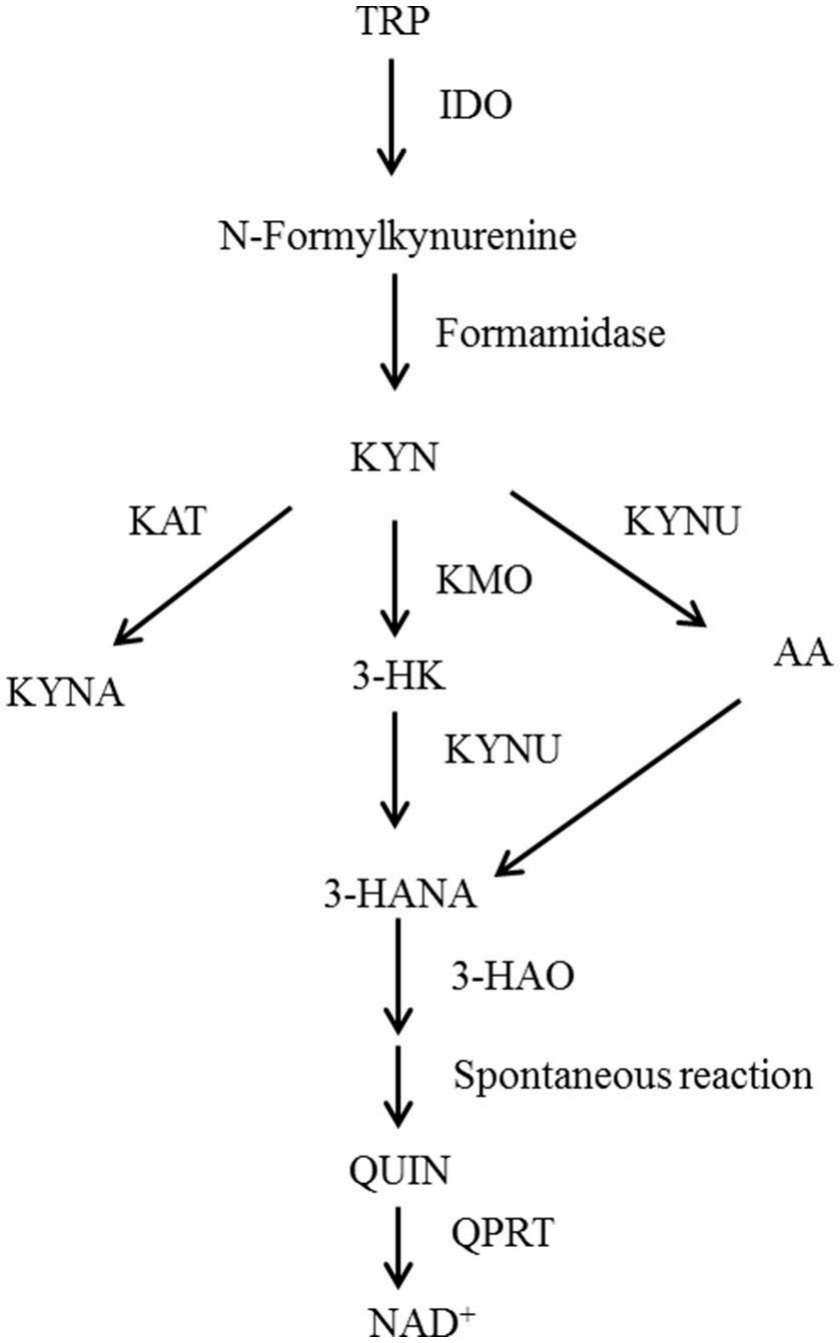
Schematic representation of the tryptophan metabolism pathway. TRP, Tryptophan; IDO, Indoleamine-2,3-dioxygenase; KYN, Kynurenine; KAT, Kynurenine aminotransferase; KMO, Kynurenine 3-monoxygenase; KYNU, Kynureninase; 3-HK, 3-Hydroxykynurenine; AA, Anthranilic acid; 3-HANA, 3-Hydroxyanthranilic acid; KYNA, Kynurenic acid; 3-HAO, 3-Hydroxyanthranilic acid oxygenase; QUIN, Quinolinic acid; QPRT, Quinolinate phosphoribosyl transferase; NAD, nicotinamide adenine dinucleotide.

Recently, researchers have found that depressive behaviors induced by cytokines are probably related to the activation of the kynurenine pathway (KP). Studies based on animal models of depressive symptoms have reported that indoleamine 2, 3-dioxygenase (IDO) is likely to be a key metabolic enzyme in the KP (8–11). Other studies have explored the metabolic processes of the KP (Figure 1). Under physiological conditions, tryptophan is transformed into kynurenine (KYN) through tryptophan 2, 3-dioxygenase. Then, KYN continues to be tabolized mainly along two independent branches. The first is a neurotoxic branch, and KYN is transformed steeply into 3-hydroxy kynurenine, 3-hydroxyo-o-aminobenzoic acid and quinolinic acid by virtue of various enzymes (e.g., canine urine-3-monooxygenase). The second is a neuroprotective branch, where KYN is transformed into kynurenic acid by kynurenine amino transferases.

Another body of research has suggested that 5-hydroxytrypine (5-HT), which is essential to emotion regulation, decreases after immune activation and this causes depression (12–14). However, other researchers have proposed that metabolic factors of the KP have neurotoxic or neuroprotective effects that damage central neuroplasticity and induce depression. Myint and Kim's widely-accepted “KP metabolic imbalance hypothesis” proposes that metabolic imbalance is the major mechanism of KP induced-depression (14–18). In line with this, variations in metabolic factors in the KP and relevant enzymes have been found in MDD, bipolar affective disorder and several neurodegenerative diseases (19, 20).

The activity of the metabolic enzyme IDO can be evaluated using the ratio KYN/TRP (21). TRP is converted to KYN by TDO or IDO. Intriguingly, the level of both of them has been found to reduce or increase in primary studies of MDD (22–25). KYN is transformed into KYNA through the metabolic enzyme KAT, which is a neuroprotective branch of the KP. KYNA is a non-selective competitive ionic glutamate receptor antagonist inhibiting the toxicity of excitatory amino acids by blocking N-methyl-d-aspartate receptor (NMDARs) and also antagonizing the α 7 nicotinic acetylcholine receptor to inhibit the release of presynaptic glutamate (19). Reduction of KYNA found in the blood and cerebrospinal fluid of MDD patients has been related to degree of depression (18, 24). The KYNA/KYN ratio could be also employed to evaluate enzyme activity of KAT. Several metabolic factors in KYN transformed to QUIN branches have been corroborated to be neurotoxic and called the neurotoxic branch of the KP, among which 3-HK could induce apoptosis (26). Furthermore, QUIN exerts a neurotoxic role through activating NMARDs. The ratio of neuroprotective factors to neurotoxic factors (KYNA/QUIN) could also be used to estimate the imbalance of the KP metabolism to assess the degree of nerve injury (27).

The theoretic basis of the KP metabolism imbalance's role in depression is derived from preclinical research focused on depression induced by immune response. However, the actual impacts of metabolic factors in the KP on primary MDD are largely elusive.

In this study, we aimed to establish if level of metabolic factors, determined by mass spectrometer analysis of peripheral blood could be used to differentiate hospital patients with depression and bipolar disorder from each other and from healthy controls. In addition we aimed to establish the association between levels of metabolic factors and severity of depression among MDD patients, and the utility of metabolic factor levels for clinical diagnosis of depression.

## Materials and methods

### Study population

Inclusion criteria for the MDD patient group included: (1) Meeting the DSM-V diagnostic criteria for MDD; (2) A of 20 or more on the HAMD-24; (3) A negative score on the Mood disorder scale (MDQ); (4) A negative screening on the Brief Screening Scale for Dementia (BSSD); (5) No psychoactive drugs taken within 1 week; (6) Han nationality, right handedness, age between 18 and 60 years. Inclusion criteria the BD patient group included: (1) Meeting the DSM-V depression diagnostic criteria for BD of DSM-V; (2) A score of 20 or more on the HAMD-24 (3) Screening positive on the MDQ; (4) Negative screening for the BSSD; (5) A score of <10 on the Young Mania Rating Scale (YMRS); (6) No psychoactive drugs taken within 1 week; (7) Han nationality, right handedness, age between 18 to 60 years. Inclusion criteria for the healthy control group included: (1) No mental illness or history of mental illness; (2) No family history of psychosis; (3) A score of <7 on the HAMD-24; (4) Negative screening on both the MDQ and BSSD; (5) Han nationality, right handedness, aged between 18 to 60 years.

Exclusion criteria for the MDD and BD groups were: (1) Somatic disease, brain organic disease or depressive disorder caused by substance abuse or dependence; (2) Personality disorder and mental retardation; (3) Bipolar attack type, rapid circulation type; (4) Major physical diseases such as heart, brain, lung, liver, kidney, or drug dependence; (5) Autoimmune diseases, chronic inflammation, severe metabolic syndrome and other diseases affecting the immune system metabolism; (6) Taking anti-inflammatory drugs, antiviral drugs, antibiotics or immunoregulatory drugs; (7) Being pregnant or lactating. Exclusion criteria for the healthy control group included: (1) A major physical disease such as heart, brain, lung, liver, or kidney disease, or drug dependence; (2) Autoimmune diseases, chronic inflammation, severe metabolic syndrome and other diseases affecting the immune system metabolism; (3) Taking anti-inflammatory drugs, antiviral drugs, antibiotics or immunoregulatory drugs; (4) Being pregnant or lactating.

After screening and assessment, 33 patients with MDD and 20 patients with BD receiving either inpatient or outpatient care at the Shanghai Mental Health Center were enrolled into the study between May 2017 to January 2018 In addition, 23 healthy volunteers who satisfied the inclusion and exclusion criteria above were recruited online between July 2017 and January 2018 into a control condition.

This study protocol was reviewed and approved by the Ethics Committee of Shanghai Mental Health Center (batch No.:2015Ky-03). All subjects voluntarily participated and signed informed consent.

### Method

#### Demographic survey, clinical evaluation, and blood collection

##### Clinical evaluation

Enrolled patients and the healthy controls were diagnosed and reviewed by one attending physician and one (deputy) chief physician according to DSM-5 criteria. All participants were assessed with the HAMD-24, MDQ, BSSD and YMRS, respectively. An interview was conducted between a member of the research team and study participants to record demographic data, including sex, age, education level, family history, and somatic diseases. Other data includes initial age of incidence, total course of disease, times of incidence, course of previous treatment.

##### Plasma sample collection

Five milliliter empty stomach venous blood at early morning was collected from participants. The venous blood was placed in an EDTA anticoagulant tube. The plasma was separated at the rate of 3,000 rpm at normal temperature in 30 min and then the plasma was stored in a tube at −80°C. Clinical evaluation was performed on the same day as blood collection.

#### Main instruments and reagents

##### Instruments

UPLC-3Q-MS (Model: ACQUITY UPLC & SCIEX SelexION Triple Quad 5500 System, America Waters and AB Sciex company); Thermo Hypersil GOLD PFP (2.1^*^100 mm 1.9 μm) liquid chromatography column (America Thermo Scientific Company); Analyst 1.5.2 chromatography-mass spectrometry control software (AB Sciex company); 0.45 μm filter membrane and its filter extractor (America, Millipore company); Low temperature bench centrifuge (America, Eppendorf); MX5 million molecular balance (Switzerland, Mettler-Toledo).

##### Reagent

standard regent of TRP, KYN, KYNA, QUIN (sigma); Methanol, acetonitrile ammonium acetate, and glacial acetic acid (Shanghai Anpu Experimental Technology Company).

#### Laboratory detection

##### Standard preparation

0.901 mg TRP, 1.248 mg KYN, 1.282 mg QUIN were weighed accurately and then dissolved and mixed in 50% methanol solution, respectively, to make 1 mg/ml standard stock solution; 0.671 mg KYNA was weighed accurately and then dissolved and mixed in ultra-pure water to make 0.167 mg/ml standard stock solution, which was split storing in −20°C. Another mixed standard working solution in 1 ug/ml was used to prepare standard curve point of concentration of 5, 10, 50, 100, 250, 500 ng/ml as well.

##### Plasma sample treatment

Plasma samples were redissolved and 50 ul plasma was placed in a 1.5 ml EP tube, then 200 ul methanol solution was added to the plasma sample. After that protein was gently shaken for 3 min on the swirl mixer of which supernatant was centrifuged at 4°C for 5 min at the rate of 12,000 rpm. Finally, collected supernatant was put into UPLC-3Q-MS instrument for analysis and detection.

Analyses were carried out using an ultra-performance liquid chromatography machine (ACQUITY UPLC; Waters, Milford, MA, USA) coupled with a triple quadrupole mass spectrometer (SCIEX SelexION Triple QuadTM 5500 System; Applied Biosystems, AB SCIEX, Foster City, CA, USA; UPLC-3QMS). Separation was obtained with a Thermo Hypersil GOLD PFP column (2.1^*^100 mm 1.9 μm) at ambient temperature using 20 mM ammonium acetate (eluent A) and acetonitrile (eluent B) as the mobile phase at a constant flow rate of 0.4 mL min-1. The eluted with the following gradient profile: 0–0.5 min: 99% A; 0.5–1.0 min: 99–65% A; 1.0–3.0 min: 65–55% A; 3.0–3.4 min: 55–2% A; 3.4–5 min 2% A, 5.0–5.1 min: 2–99% A; 5.1–7.0 min: 99% A. The electron spray ionization (ESI) source used in this study was operated in positive ion mode, and its main working parameters were as follows: ion spray voltage, 5,500 V; curtain gas, 35 psi; both GS1 (Nebulizer Gas) and GS2 (Heater Gas), 55 psi; and probe temperature, 600°C. The declustering potential (DP), entrance potential (EP), collision cell exit potential (CXP), and collision energy (CE) values were used to perform multiple reaction monitoring (MRM) and were optimized automatically by the software for each of the analytes (Table 1).

**Table 1:** Detection parameters of UPLC-3Q-MS.

**Q1**	**Q3**	**DP(V)**	**ID**	**CE(V)**
205.1	188.2^*^	40	Trp	13
205.1	118.2	40	Trp	34
209.1	192.1^*^	40	Kyn	12
209.1	94	40	Kyn	17
190	144.2^*^	60	Kyna	22
190	89	60	Kyna	50
168	124^*^	60	QUIN	13
168	78	60	QUIN	28

*Q1, parient ion; Q2, daughter ion; ID, identification; DP, decluster voltage; CE, collision voltage*;

* *, quantitative ion*.

##### Qualitative and quantitative analysis of metabolic factors

Peak retention value comparison method and superposition method are employed to analyzed TRP, KYN, KYNA, and QUIN qualitatively. The peak area was determined quantitatively by external standard method. Acquisition and processing of chromatographic data was accomplished by Analyst 1.5.1. Concentration of plasma metabolic factor (ng/ml) = peak area of metabolic factor in plasma/peak area of metabolic factor in standard solution ^*^concentration of metabolic factor in standard solution^*^2.

#### Statistical analysis

All data in this study were statistically analyzed by SPSS19.0 and MedCalc software. Age, education years, course of disease, first onset age, HAMD-24 scores and level of metabolic factors were positively distributed, presented by mean ± standard deviation, incidence times were measurement data with non-normal distribution, presented by quartile; sex, and family history are presented by frequency; Comparison of measurement data with positive distribution among groups was analyzed by one way-ANOVA; *t*-test was used to analyze normal distribution data between two groups, frequency data is analyzed by chi-square test; Correlation between HAMD-24 score and metabolic factor level is analyzed by Pearson correlation test; ROC curve was used to evaluate the efficacy of metabolic factor level in diagnosing depression. A significance level of 0.05 was used.

## Results

### General demographic data

Characteristics of each group are presented in Table 2. There were no significant differences between groups on demographic variables including age, gender and level of education. First onset age in the MDD group was higher than in the BD group (see Table 2). Episode duration, course of disease, and family history were not significantly different across the depression groups (see Table 2).

**Table 2:** Demographic data among three groups.

**Characteristic**	**MDD (*n* = 33)**	**BD (*n* = 20)**	**HC (*n* = 23)**	***T/F/X^2^***	***P***
Age (year)	28.39 ± 5.20	30.45 ± 5.08	29.30 ± 5.89	0.912	0.406
Gender (male/female)	10/23	8/12	12/11	2.717	0.257
Education year (year)	12.36 ± 2.15	11.85 ± 1.76	12.09 ± 2.81	0.326	0.723
HAMD scores	31.73 ± 9.93	32.65 ± 11.08	0.00 ± 0.00	109.761	<0.001^*^
Incidence times	2 (1,4)	2.5 (1.25,4.5)	–	0.386	0.700
Course of disease (month)	96.00 ± 88.82	97.73 ± 124.09	–	0.059	0.953
First onset age (year)	36.67 ± 17.21	22.35 ± 6.22	–	4.335	<0.001^*^
Family history (%)	8/24.24	8/40.00	–	1.467	0.355

*MDD, major depressive disorder; BD, bipolar depression; HC, healthy control; HAMD, Hamilton Depression Rating Scale*;

* *P < 0.05; –, no relevant data*.

### KP metabolic factor analysis

**Figure 2:**
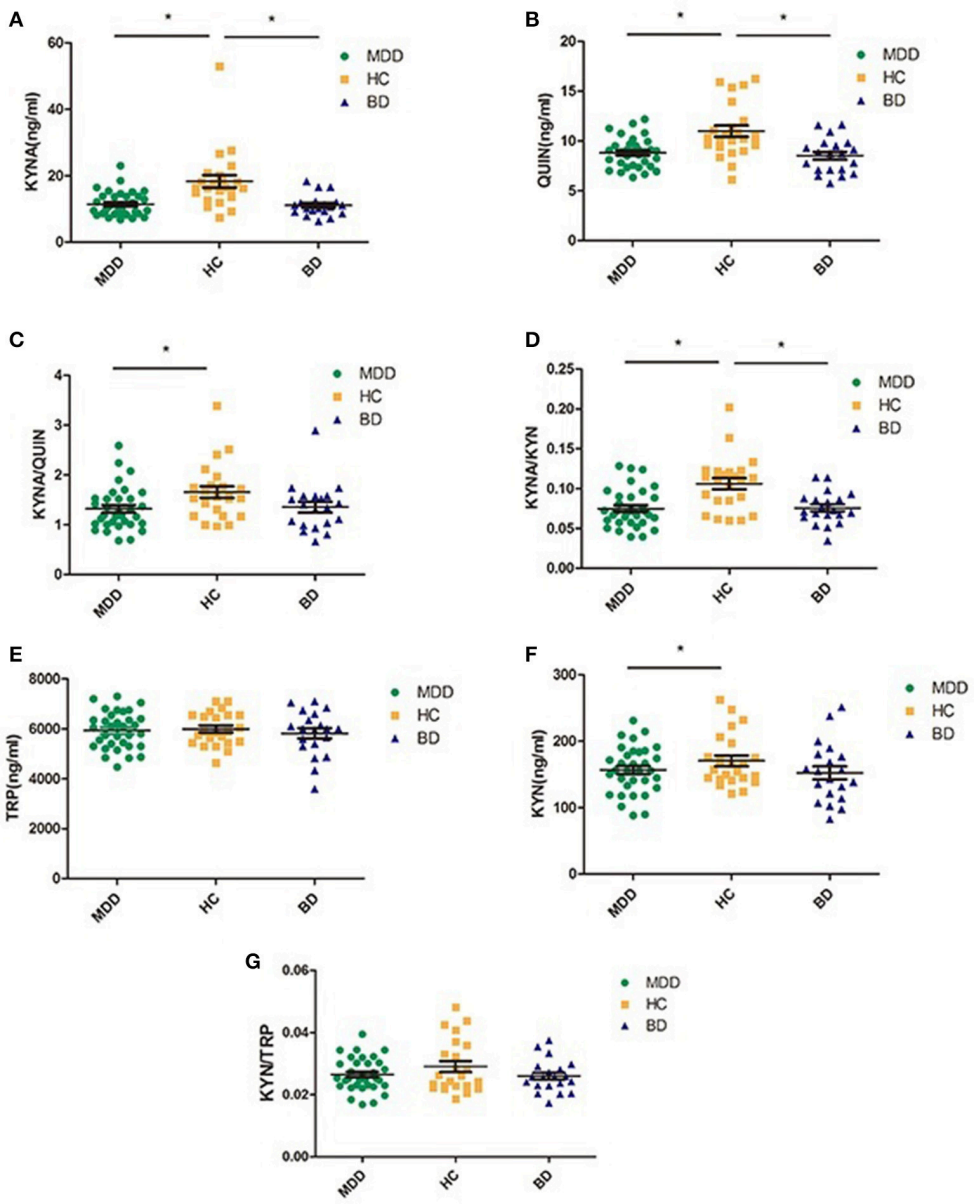
Comparison of various metabolic factors in KP among three groups (MDD, BD, and HC); **(A)** compared with HC group, both MDD and BD groups showed significantly lower KYNA levels; **(B)** compared with HC group, both MDD and BD groups showed significant lower QUIN levels, **(C)** the index of KYNA/QUIN was significantly lower in the MDD group than in the HC group; **(D)** the index of KYNA/KYN was significantly lower in both the MDD and the BD groups than HC group, **(E)** there was no significant difference among the three groups in TRP levels; **(F)** compared with the HC group, the MDD group showed significantly lower KYN levels; **(G)** there was no significant difference among three groups. MDD, major depressive disorder; BD, bipolar disorder; HC, healthy controls; TRP, Tryptophan; KYN, Kynurenine; KYNA, Kynurenic acid; QUIN, Quinolinic acid; *P < 0.05.

Table 3 and Figure 2 present levels of metabolic factors for the three groups. ANOVA revealed that there was a significant group effect for KYNA (*F* = 11.941, *P* < 0.001) and QUIN (*F* = 10.752, *P* < 0.001), KYNA/QUIN (*F* = 3.508, *P* = 0.035), and KYNA/KYN (*F* = 10.857, *P* < 0.001). There was no group effect for TRP, KYN, or KYN/TRP.

**Table 3:** Comparison of metabolic factors in the KP among three groups.

**Metabolic factors**	**MDD^a^(*n* = 33)**	**BD^b^(*n* = 33)**	**HC^c^(*n* = 23)**	***F***	***P***	**Bonferroni group comparison**
TRP (ng/ml)	5933.64 ± 740.19	5821.00 ± 912.81	5995.22 ± 667.65	0.270	0.764	ns
KYN (ng/ml)	156.76 ± 35.47	152.45 ± 44.90	170.50 ± 39.55	1.299	0.279	ns
KYNA (ng/ml)	11.43 ± 3.73	11.09 ± 3.10	18.32 ± 9.02	11.941	<0.001^*^	c>a;c>b;
QUIN (ng/ml)	8.80 ± 1.49	8.52 ± 1.73	11.00 ± 2.73	10.752	<0.001^*^	c>a;c>b;
KYNA/QUIN	1.32 ± 0.44	1.35 ± 0.48	1.66 ± 0.56	3.508	0.035^*^	c>a;c>b;
KYN/TRP	0.0264 ± 0.0053	0.0259 ± 0.0052	0.0290 ± 0.0085	1.519	0.226	ns
KYNA/KYN	0.075 ± 0.024	0.076 ± 0.020	0.106 ± 0.034	10.857	<0.001^*^	c>a;c>b;

*MDD, major depressive disorder; BD, bipolar depression; HC, healthy control; TRP, Tryptophan; KYN, Kynurenine; KYNA, Kynurenic acid; QUIN, Quinolinic acid; ns: no significant difference at the levels of p < 0.05*.

a *MDD*;

b *D*;

c *HC*;

* *P < 0.05*.

*Post hoc* testing revealed that MDD patients had lower levels of KYNA, QUIN, KYNA/QUIN, and KYNA/KYN than those in the HC group (all *P* < 0.05). BD patients also had lower levels of KYNA, QUIN, and KYNA/KYN than healthy controls (*P* < 0.05). There were no significant differences in the metabolic factors between the MDD and BD patients (see Figure 2).

### Correlation analysis between baseline KP metabolic factors and HAMD-24 scores

**Figure 3:**
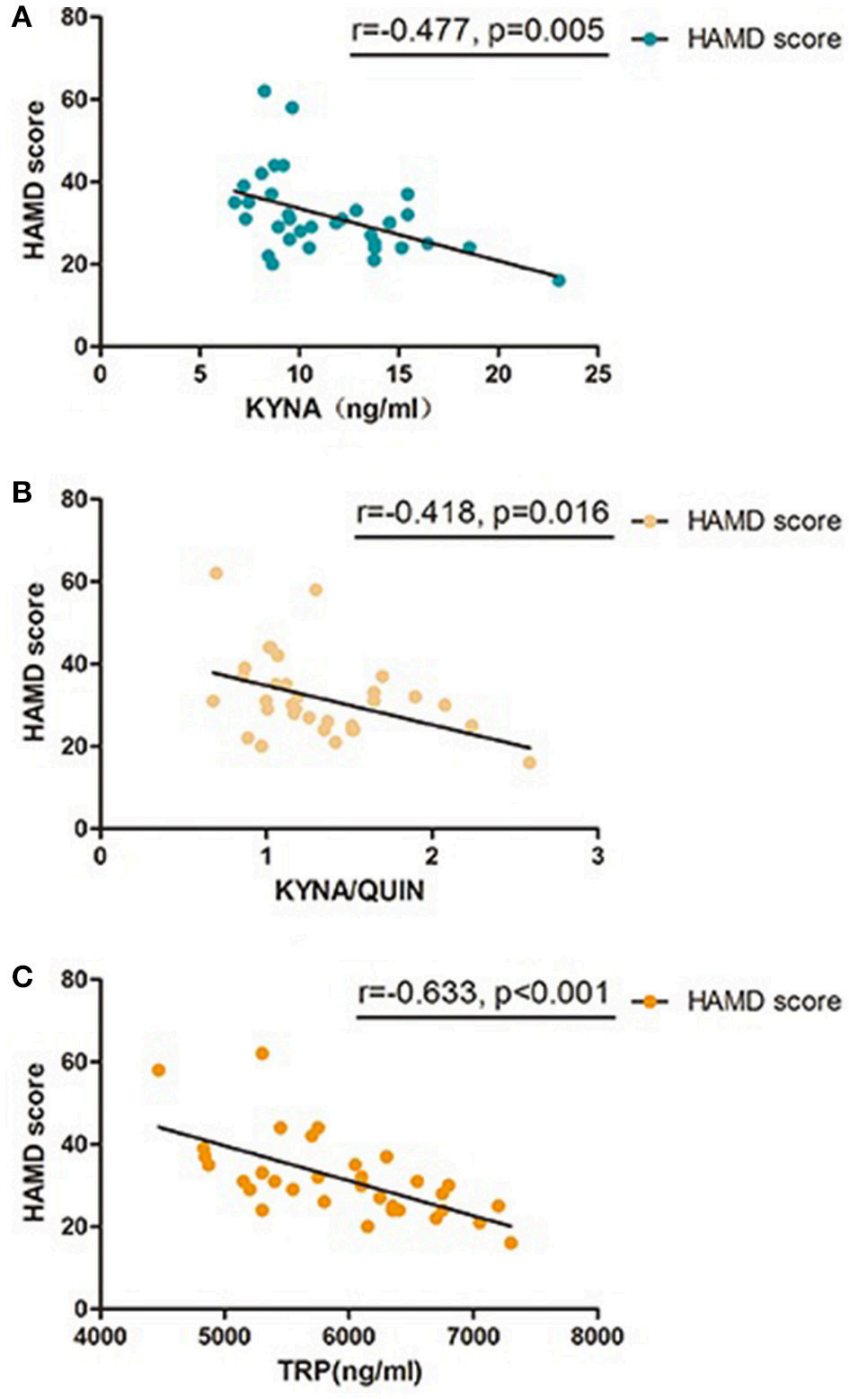
Pearson correlation between baseline KP metabolic factors and HAMD-24 scores in MDD group, **(A)** KYNA was significant correlated with HAMD-24 scores in patients with MDD; **(B)** KYNA/QUIN was significant correlated with HAMD-24 scores in patients with MDD; **(C)** TRP was significant correlated with HAMD-24 scores in patients with MDD; MDD, major depressive disorder; TRP, Tryptophan; KYN, Kynurenine; KYNA, Kynurenic acid; QUIN, Quinolinic acid; HAMD, Hamilton Depression Rating Scale.

To further investigate the association between KP and MDD, Pearson correlation analysis was performed between levels of metabolic factors and HAMD scores which are closely related to the severity of MDD. Results show that HAMD-24 scores were negatively correlated with TRP, KYNA, and KYNA/QUIN levels (*r* = −0.633, *P* < 0.001; *r* = −0.477, *P* = 0.005; *r* = −0.418, *P* = 0.016, respectively, see Table 4 and Figure 3). Of the metabolic factors, TRP had the strongest association with severity of depression.

**Table 4:** Correlation between HAMD scores and metabolic factors of KP in MDD.

**Metabolic factors**	***r*-value**	***P***
TRP (ng/ml)	−0.633	<0.001^*^
KYN (ng/ml)	−0.243	0.173
KYNA (ng/ml)	−0.477	0.005^*^
QUIN (ng/ml)	−0.033	0.854
KYNA/QUIN	−0.418	0.016^*^
KYN/TRP	0.117	0.516
KYNA/KYN	−0.218	0.222

*TRP, Tryptophan; KYN, Kynurenine; KYNA, Kynurenic acid; QUIN, Quinolinic acid*;

* *P < 0.05*.

### Metabolic factors of KP in diagnosing MDD

**Figure 4:**
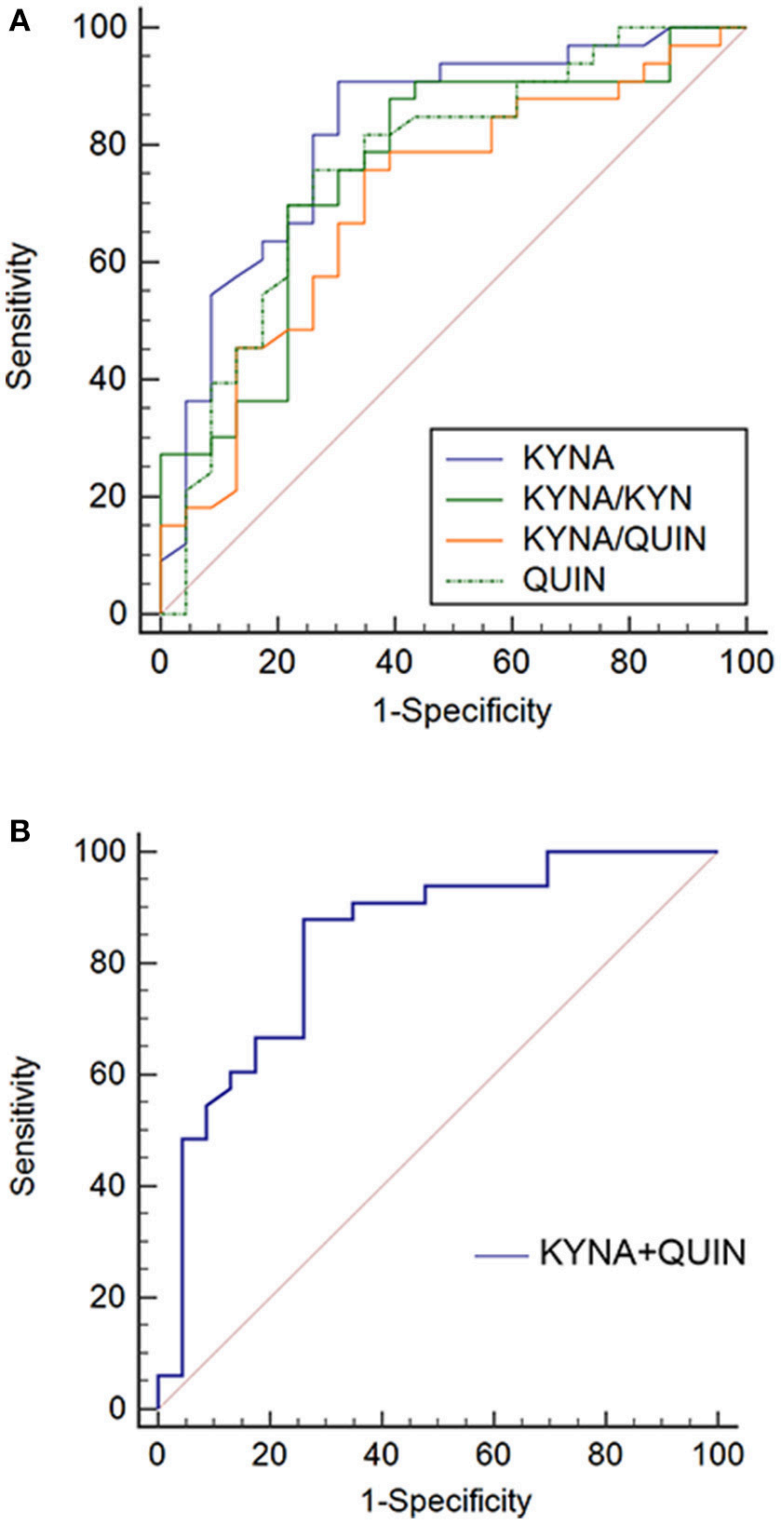
ROC curves of metabolic factors in MDD. **(A)** Diagnosis accuracy of KYNA, QUIN, KYNA/QUIN, and KYNA/KYN in MDD: 82.5, 76.6, 70.6, 76.0%, respectively; **(B)** ROC curve of combination of KYNA and QUIN, the combination diagnosis accuracy reached to 83.6%. MDD, major depressive disorder; TRP, Tryptophan; KYN, Kynurenine; KYNA, Kynurenic acid; QUIN, Quinolinic acid.

Given that the previous analyses demonstrate an association between metabolic factors of KP and MDD, ROC analysis was performed to establish a diagnostic value of these metabolic factors. Accuracy of KYNA, QUIN, KYNA/QUIN, KYNA/KYN was 82.5, 76.6, 70.6, 76.0%, respectively, among which KYNA was the highest with an optimal diagnostic value 15.45 ng/ml. Sensitivity and specificity were 90.9 and 69.6%, respectively (see Figure 4). Given that KYNA and QUIN have higher accuracy, combination analysis of KYNA and QUIN was performed and diagnostic accuracy reached 83.6%.

## Discussion

We found variations of metabolic factors in the KP existed between healthy controls and both MDD and BP. However, there were no significant differences between MDD and BD. Several of these metabolic factors were shown to be intimately correlated to severity of depression in the MDD group. Furthermore, QUIN, ratio of KYNA/QUIN and KYNA/KYN were found to be potential biomarkers for diagnosing MDD. Intriguingly, our results indicated that KYNA could be a bona-fide biomarker for diagnosing MDD with an accuracy of 82.5%.

### Variations of KP-related metabolic factors in depressive patients

Our results show that levels of KYNA, QUIN, KYNA/KYN, KYNA/QUIN in MDD pateints are lower compared to healthy controls. Previous studies have shown that KYNA has neuroprotective effects by blocking toxicity of excitatory amino acids, but is lower in MDD patients (19). In addition, the activity of KAT is reduced in light of decreased metabolism from the KP to the neuroprotective branch resulting in a decrease of KYNA/KYN (18, 24, 28), and KYNA/QUIN has been reported to be a crucial indicator of metabolic imbalance of the KP (29). Our results are consistent with these findings. In addition, variations in these metabolic factors between MDD patients and healthy controls also provide indications for clinical diagnosis of depression. However, the relative decrease in QUIN level was not consistent with the conclusion that QUIN level increases exert a neurotoxic role by activating NMDARs when depression occurs (16, 30). This could be due to the fact that QUIN is not the final product of the KP and would continue to degrade to NAD causing QUIN levels to decrease (31). In the future, it is recommended that neurotoxic factors and relative enzymes such as 3-HK and 3-HANA be measured in the KP toxic branch to evaluate downstream activity.

Consistent with research that found no evident variation of TRP and KYN in mood disorder patients compared to control controls (32), our results also showed that there was no significant difference in KYN, TRP, and KYN/TRP in the MDD group compared to the healthy control group. It is widely accepted that KYN/TRP, representing enzymatic activity of IDO, would elevate under inflammatory conditions activated by cytokines. However, this was not conformed by our results. It is possible that metabolic variation of the KP was induced by non-inflammatory activation. For example, neurotoxicity transformation of several metabolic factors or metabolic enzymes could have been induced by genetic or epigenetic changes (33). Or, variation of ratio could be due to KYN/TRP being co-regulated by both IDO and TDO (34).

### Correlation between metabolic factors and severity of depression

We used HAMD-24 scores as an indication of the severity of depression, and found that for the MDD patient group they were negatively correlated with levels of TRP, KYNA, and KYNA/QUIN. This suggests that the severity of depression could be intimately related to them, with lower levels of these metabolic factors being related to more severe symptoms of depression. If this is the case, patients with lower levels of metabolic factors should get more attention from clinical staff and superior nursing care. Among these factors, TRP has the strongest association with depression severity (*r* = 0.633). Given that there was no remarkable variation between MDD and control on this factor, TRP could exert a crucial role in depression remission, which is consistent with previous studies (35, 36) and could be further explored through expanding objects and establish animal model.

### Clinical diagnostic value of KP in MDD

Finally, given that metabolic factors varied between the MDD and healthy control groups, we analyzed the diagnostic value of metabolic factors by ROC curve. Previous studies have shown that various inflammatory factors could be diagnostic indicators for MDD, while their accuracy and stability was yet to be determined (37–39). The results showed that the diagnostic value of KYNA, QUIN, KYNA/QUIN, and KYNA/KYN all exceeded 70%, with KYNA being the highest at 82.5%. Compared to QUIN, which could be further degraded, KYNA had higher stability supporting its excellent accuracy. Further, in light of the ratio value being affected by more elements, the ratios of KYNA/QUIN and KYNA/KYN were lower. The sensitivity and specificity of the KYNA indicator were found to be 90.9 and 69.6%, respectively, which suggests that KYNA could be used to screen clinical depressive patients and healthy people due to its high sensitivity. The lower specificity suggests that patients screened positive should be further assessed. We also found that the diagnostic accuracy of the metabolic factors reached 83.6% after KYNA and QUIN were combined (16, 19), which indicated sensitivity and specificity could be elevated through combination. An explanation for this could be that KYNA and QUIN exert neuroprotective and neurotoxic role separately in the KP, representing various branches of the KP. A combination of both of them would thus realize a higher diagnostic accuracy. These results suggest that metabolic factors of the KP could be potential biomarkers for diagnosing depression and that KYNA is likely to be the most valuable. Studies with larger samples may identify a more precise cut-off value.

### Clinical significance

Our investigation probed deeper into the KP metabolic factors in MDD and BD than previous studies, and provides more detailed evidence for variations of metabolic factors of the KP in MDD or BD. The associations between HAMD-24 scores and metabolic factors in MDD suggest variations of in TRP exert a critical role in disease remission. Most importantly, KYNA is a potential biomarker discriminating MDD and healthy controls, offering a novel diagnostic method for clinicians.

## Author contributions

HL and DP conceived and designed the experiments. HL, LD, HZ, HW, DZ, and CW collected and evaluated study samples. HL, ZL, and JY performed the experiments. HL analyzed the data. HL and LD wrote the manuscript. DP, DM, and HZ edited the manuscript.

### Conflict of interest statement

The authors declare that the research was conducted in the absence of any commercial or financial relationships that could be construed as a potential conflict of interest.
